# Longitudinal Associations of Plasma Phospholipid Fatty Acids in Pregnancy with Neonatal Anthropometry: Results from the NICHD Fetal Growth Studies—Singleton Cohort

**DOI:** 10.3390/nu14030592

**Published:** 2022-01-29

**Authors:** Emily Wang, Yeyi Zhu, Rana F. Chehab, Jing Wu, Stefanie N. Hinkle, Natalie L. Weir, Andrew A. Bremer, Jiaxi Yang, Zhen Chen, Michael Y. Tsai, Cuilin Zhang

**Affiliations:** 1 Department of Epidemiology & Biostatistics, University of California, Berkeley, CA 94704, USA; emilyzwang@berkeley.edu; 2 Division of Research, Kaiser Permanente Northern California, Oakland, CA 94612, USA; yeyi.zhu@kp.org (Y.Z.); rana.chehab@kp.org (R.F.C.); 3 Department of Epidemiology & Biostatistics, University of California, San Francisco, CA 94158, USA; 4 Glotech Inc., Bethesda, MD 20850, USA; jing.wu2@nih.gov; 5 Department of Biostatistics, Epidemiology and Informatics, Perelman School of Medicine, University of Pennsylvania, Philadelphia, PA 19104, USA; stefanie.hinkle@pennmedicine.upenn.edu; 6 Division of Intramural Population Health Research, *Eunice Kennedy Shriver* National Institute of Child Health and Human Development, National Institutes of Health, Bethesda, MD 20817, USA; chenzhe@mail.nih.gov; 7 Department of Laboratory Medicine and Pathology, University of Minnesota, Minneapolis, MN 55455, USA; weirx065@umn.edu (N.L.W.); tsaix001@umn.edu (M.Y.T.); 8 Pediatric Growth and Nutrition Branch, *Eunice Kennedy Shriver* National Institute of Child Health and Human Development, National Institutes of Health, Bethesda, MD 20817, USA; andrew.bremer@nih.gov; 9 Department of Epidemiology, Harvard T. H. Chan School of Public Health, Boston, MA 02115, USA; jiaxiyang@g.harvard.edu; 10 Epidemiology Branch, Division of Population Health Research, *Eunice Kennedy Shriver* National Institute of Child Health and Human Development, National Institutes of Health, Bethesda, MD 20817, USA; 11 Department of Obstetrics & Gynaecology, Yong Loo Lin School of Medicine, National University of Singapore, Singapore 117597, Singapore; 12 NUS Bia-Echo Asia Centre for Reproductive Longevity and Equality (ACRLE), Yong Loo Lin School of Medicine, National University of Singapore, Singapore 117597, Singapore

**Keywords:** neonatal anthropometric measures, longitudinal, *n*-3 polyunsaturated fatty acids, *n*-6 polyunsaturated fatty acids, objective assessment, pregnancy

## Abstract

Despite increasing interest in the health effects of polyunsaturated FAs (PUFAs), their roles in fetal and neonatal growth remain understudied. Within the NICHD Fetal Growth Studies—Singleton Cohort, we prospectively investigated the associations of individual and subclasses of plasma phospholipid PUFAs at gestational weeks (GW) 10–14, 15–26, 23–31, and 33–39 with neonatal anthropometric measures as surrogates for fetal growth among 107 women with gestational diabetes mellitus (GDM) and 214 non-GDM controls. Multivariable weighted linear regression models estimated the associations between plasma phospholipid PUFAs and neonatal anthropometric measures. Adjusted beta coefficients for phospholipid docosahexaenoic acid (DHA) per standard deviation (SD) increase at GW 23–31 in association with birthweight z-score, neonatal length, and neonatal fat mass were 0.25 (95% CI: 0.08–0.41), 0.57 (0.11–1.03) cm, and 54.99 (23.57–86.42) g, respectively; all false discovery rates (FDRs) < 0.05. Estimated Δ5-desaturase activity per SD increase at GW 33–39 but not at other time points was positively associated with birthweight z-score: 0.29 (95% CI: 0.08–0.33); neonatal length: 0.61 (0.29–0.94) cm; and neonatal fat mass: 32.59 (8.21–56.96) g; all FDRs < 0.05. Longitudinal analysis showed consistent results. Our findings suggest that mid-to-late pregnancy presented as critical windows for primarily diet-derived DHA and Δ5-desaturase activity in relation to neonatal anthropometric measures.

## 1. Introduction

Optimal intake of nutrients during pregnancy is important not only for growth and development in utero but also in the prenatal period and later life [1,2,3]. Despite the fact that maternal glucose is the dominant fuel source for the fetus [4], it is not the sole driver of fetal development [5]. Other contributors, such as maternal lipids, are available to the fetus due to the presence of placental lipoprotein receptors, lipoprotein lipase, and fatty-acid-binding protein, suggesting the need to further evaluate the role of diverse maternal fuels in birth outcomes [6,7].

Despite increasing interest in the health benefits of polyunsaturated FAs (PUFAs) [8], the roles of the two classes of plasma phospholipid PUFAs, *n*-3 and *n*-6 PUFAs, in fetal growth and development remain understudied. In addition, emerging evidence indicates that individual phospholipid FAs may have distinct nutritional and physiologic roles in human metabolism [9,10,11,12]. Prenatal PUFA concentrations have been linked to the duration of pregnancy, neonatal respiratory distress syndrome, and childhood adiposity [13,14,15]. To date, data on the associations of maternal plasma phospholipid PUFA composition in pregnancy with neonatal birth size and adiposity have been limited and inconsistent [16,17]. Further, nascent evidence suggests that fetal growth may be influenced by maternal lipids to a different extent depending on the timing of exposure during gestation [18,19,20]. However, previous data on maternal lipid levels and fetal growth have been limited to cross-sectional studies with a one-point-in-time measurement of triglyceride levels, mostly in late pregnancy [21,22,23]. As such, longitudinal data on maternal individual phospholipid PUFA concentrations throughout pregnancy are needed to improve our understanding of the impact of intrauterine nonglycemic nutrient supplies on neonatal anthropometric measures as surrogate measures for intrauterine growth and fetal development.

To address these knowledge gaps, we aimed to investigate the longitudinal and prospective associations of individual and subclasses of plasma phospholipid PUFA levels throughout pregnancy with neonatal anthropometric measures and to explore the sensitive window of exposure to plasma phospholipid PUFAs in pregnancy in relation to neonatal size and adiposity.

## 2. Materials and Methods

### 2.1. Study Sample

This study used data from low-risk pregnancies within the *Eunice Kennedy Shriver* National Institute of Child Health and Human Development (NICHD) Fetal Growth Study—Singleton Cohort (approval code: 09-CH-N152) [24,25]. The study enrolled a total of 2802 pregnant women in gestational weeks 8–13 at 12 U.S. clinical centers from 2009 to 2013. The study recruited pregnant women aged 18–40 years who were free of preexisting diseases, such as hypertension, diabetes mellitus, cancer, human immunodeficiency virus, or acquired immunodeficiency syndrome. All participating clinical sites and the NICHD obtained approval from their respective institutional review boards. All participants provided written and informed consent.

This study used biomarker data measured in a nested case–control study, the primary aim of which was to assess the associations between biomarkers in early to mid-pregnancy and the subsequent risk of gestational diabetes (GDM) [12]. The secondary aim of the GDM case–control study was to assess associations between biomarkers and fetal growth, as assessed by neonatal anthropometric measures. This study leveraged biomarker data in 321 women (107 GDM cases and 214 non-GDM controls) from the GDM case–control study. The GDM cases were identified via medical record review according to the Carpenter and Coustan criteria [26]. Each GDM case was individually matched 1:2 to non-GDM controls (*n* = 214) by age (±2 years), race/ethnicity (non-Hispanic White, non-Hispanic Black, Hispanic, and Asian/Pacific Islander), and gestational age at blood sample collection (±2 weeks).

### 2.2. Maternal Biomarker Assessment

Maternal blood specimens were collected at four time points during pregnancy. Two blood samples were collected before the diagnosis of GDM, followed by one sample around and one sample after the diagnosis of GDM. We ensured at least one collection per trimester to assess the clinically relevant trimester-specific physiologic alterations throughout pregnancy. Specifically, biomarkers from the first two blood collections were measured at gestational weeks 10–14 (median 13, interquartile range (IQR) 12–14) and 15–26 (after an overnight fast of 8–14 h; median 19, IQR 18–21) among all cases (*n* = 107) and controls (*n* = 214). The two subsequent blood samples were measured at gestational weeks 23–31 (median: 27, IQR: 25–28) and weeks 33–39 (median: 36, IQR: 35–37) among cases and one of their randomly selected controls (both *n* = 107). The fasting duration prior to maternal biospecimen collection at all four visits was similar between cases and controls. The blood samples were immediately processed into plasma and stored at <−80 °C until analysis. Plasma phospholipid PUFA concentrations were measured by a Hewlett Packard 5890 gas chromatography system with flame ionization detection as described previously [12,27]. In short, we extracted total lipids from plasma and separated the phospholipid fraction by thin-layer chromatography. Isolated phospholipids were converted to fatty acid methyl esters and further separated using gas chromatography. We then used mixtures of known fatty acid methyl esters purchased from Nu-Chek Prep (Elysian Township, MN, USA) to identify fatty acids. Among 28 fatty acids, we identified 11 PUFAs, including 4 *n*-3 PUFAs—18:3*n*-3 (alpha-linolenic acid, ALA), 20:5*n*-3 (eicosapentaenoic acid, EPA), 22:5*n*-3 (docosapentaenoic acid, *n*-3 DPA), and 22:6*n*-3 (docosahexaenoic acid, DHA)—and 7 *n*-6 PUFAs: 18:2*n*-6 (linoleic acid, LA), 18:3*n*-6 (gamma-linolenic acid, GLA), 20:2*n*-6 (eicosadienoic acid, EDA), 20:3*n*-6 (dihomo-gamma-linolenic acid, DGLA), 20:4*n*-6 (arachidonic acid, AA), 22:4*n*-6 (docosatetraenoic acid, DTA), and 22:5*n*-6 (docosapentaenoic acid, *n*-6 DPA). The content of individual plasma phospholipid PUFA was expressed as a percentage (%) of the total phospholipid fatty acids. This approach has been validated and widely adopted in epidemiological studies and tends to facilitate a better interpretation of metabolic associations compared to absolute measurements [28]. Product-to-precursor PUFA ratios were derived to estimate FA elongase and desaturase enzyme activities: 18:3*n*-6/18:2*n*-6 (GLA/LA), indicating estimated Δ6-desaturase activity catalyzing the conversion of LA to GLA; 20:4*n*-6/20:3*n*-6 (AA/DGLA), indicating estimated Δ5-desaturase activity catalyzing the conversion of DGLA to AA; and 20:3*n*-6/18:2*n*-6 (DGLA/LA), indicating the conversion of LA to DGLA [29,30,31].

### 2.3. Neonatal Anthropometric Measures

Birthweight and gestational age at delivery were abstracted and calculated from neonatal medical records. Neonatal measurements were taken 12–24 h after delivery and obtained in duplicate. Infants born very preterm (≤32 weeks) and moderately preterm (33–36 weeks) were measured at 32 completed weeks of gestation-corrected age and when stabilized, respectively. Birthweight z-score was calculated based on the sex- and gestational-age-specific U.S. national reference percentiles of birthweight [32]. Neonatal length in cm, the distance from the infant’s feet to the top of the head, was measured 12–24 h after delivery by verified study personnel using an infantometer. Skinfold measurements in cm were taken using a Lange skinfold caliper on the right side of the body at the abdominal flank, anterior thigh, subscapular, and triceps. The measurements were summed as an indicator for neonatal adiposity [33]. Fat mass was estimated using the method developed by Catalano et al.: fat mass = 0.39055 × birthweight + 0.0453 × flank skinfold − 0.03237 × birth length + 0.54657 [34].

### 2.4. Covariates

Maternal demographic, lifestyle, and clinical factor data were collected from medical records and structured questionnaires. Covariates were selected a priori: maternal age (years), race/ethnicity (non-Hispanic White, non-Hispanic Black, Hispanic, and Asian), education (high school or less, some college/associate degree, and four-year college degree or higher), nulliparity (yes/no), and pre-pregnancy body mass index (underweight: < 25.0; normal: 25.0–29.9; overweight: 30.0–34.9; obese: 35.0–44.9 kg/m^2^), gestational weight gain up to the respective visit (continuous), gestational age at blood collection (weeks), gestational age at delivery (weeks), and postnatal days at neonatal assessment (continuous; for all models except birthweight). In this low-risk population, women without obesity who smoked during the 6 months preceding the index pregnancy were ineligible, and only five women with obesity reported smoking during the 6 months before pregnancy. Thus, smoking was not included as a covariate.

### 2.5. Statistical Methods

For all analyses, the matched case–control sample was reweighted to represent the entire original cohort. The sampling weights were created via an inverse likelihood approach [35]. GDM subjects had a sampling probability of 1. The sampling probability of each selected non-GDM control was calculated using logistic regression among all non-GDM women in the entire cohort, including matching factors for control selection (age, race/ethnicity, and gestational week at blood collection). We used bootstrapping with 200 replicates to confirm the variance of our weighted models and observed similar findings.

For the primary analyses, we treated sex- and gestational-age-specific birthweight z-score, neonatal length, and neonatal fat mass as continuous variables. Individual PUFAs and PUFA ratios were each analyzed as a continuous variable per standard deviation (SD). Multivariable linear regression models were fitted to assess the temporal associations of individual PUFAs, subclasses of PUFAs (i.e., *n*-3 and *n*-6 PUFAs), and PUFA ratios at each of the four visits (gestational weeks 10–14, 15–26, 23–31, and 33–39 weeks) with neonatal anthropometric measures, adjusting for the aforementioned covariates. Further, the longitudinal trends of plasma phospholipid PUFAs and ratios during pregnancy were assessed by fitting generalized linear mixed models with participant-specific random intercepts, an autoregressive covariance structure, a random effect for the matched case–control pairs, and an interaction term of a cross product between concentrations of plasma phospholipid PUFAs and sample collection time. The Benjamini–Hochberg false discovery rate (FDR)-controlling method was used as the post hoc adjustment for multiple comparisons.

We further explored whether pre-pregnancy obesity status modified the associations of individual plasma phospholipid PUFA and PUFA ratios with neonatal anthropometric measures. P for interaction was obtained using the likelihood ratio test. An interaction effect was considered significant if the *p* value was <0.10, recognizing the limitations in statistical power when testing for interactions in multiplicative models [36,37,38,39]. Models stratified by pre-pregnancy obesity status were adjusted for the aforementioned covariates, including pre-pregnancy BMI as a continuous variable to account for any residual confounding within each stratum. We conducted a sensitivity analysis by excluding the five women with obesity who reported smoking during the 6 months before pregnancy. All analyses were conducted using SAS version 9.4 (SAS Institute, Cary, NC, USA) and R version 3.0.2 (Vienna, Austria).

## 3. Results

The study sample with biomarker measurements within the NICHD Fetal Growth Studies—Singleton Cohort was socio-demographically diverse after reweighting: 30.9% of women were non-Hispanic White, 23.3% were non-Hispanic Black, 27.2% were Hispanic, and 18.5% were Asian/Pacific Islander (Table 1; see unweighted distribution in Supplemental Table S1). Approximately half of the study participants were classified as either overweight (33.1%) or obese (15.2%). All 95% confidence intervals around estimates of distributions of the reweighted sample contained the original estimate based on the entire cohort, confirming effective reweighting.

Among all plasma phospholipid PUFAs, LA (18:2*n*-6) was the most abundant form, accounting for 20.6–22.5% of the total plasma phospholipid FAs, followed by AA (20:4*n*-6; 9.8–11.2%) and DHA (22:6*n*-3; 3.9–4.2%; Table 2). All other individual PUFAs contributed <1% to the total plasma phospholipid PUFA, with the lowest concentration contributed by GLA (18:3*n*-6; 0.07–0.08%) during pregnancy. There was an overall decreasing trend for DPA, the sum of *n*-3 PUFA levels, AA, estimated Δ5-desaturase activity, and DGLA/LA (all *p* < 0.05) and no change in the sum of *n*-6 PUFA concentrations during pregnancy.

**Figure 1:**
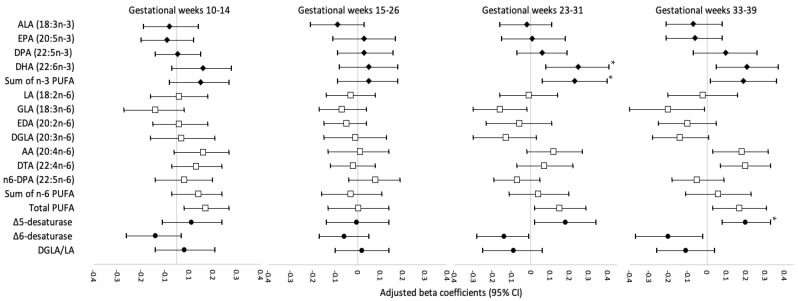
Adjusted beta coefficients and 95% CI for birthweight z-score in association with individual and subclasses of maternal plasma phospholipid PUFAs and PUFA ratios per standard deviation increase at four visits during pregnancy. AA, arachidonic acid; ALA, alpha-linolenic acid; CI, confidence interval; DHA, docosahexaenoic acid; DGLA, dihomo-gamma-linolenic acid; DPA, docosapentaenoic acid; DTA, docosatetraenoic acid; EDA, eicosadienoic acid; EPA, eicosapentaenoic acid; FDR, false discovery rate; GDM, gestational diabetes mellitus; GLA, gamma-linolenic acid; LA, linoleic acid; PUFA, polyunsaturated fatty acid; Δ5-desaturase, AA/DGLA; Δ6-desaturase, GLA/LA; Risk estimates were adjusted for maternal age (continuous), race/ethnicity (non-Hispanic White, non-Hispanic Black, Hispanic, Asian/Pacific Islander), education (high school or less, some college/associate degree, 4-year college degree or higher), nulliparity (yes/no), pre-pregnancy body mass index (<25.0, 25.0–29.9, 30.0–34.9, 35.0–44.9 kg/m^2^), gestational weight gain up to the respective visit (continuous), gestational week at blood collection (continuous), and gestational age at delivery (continuous). * Significant associations after FDR adjustment (*p* < 0.05).

**Figure 2:**
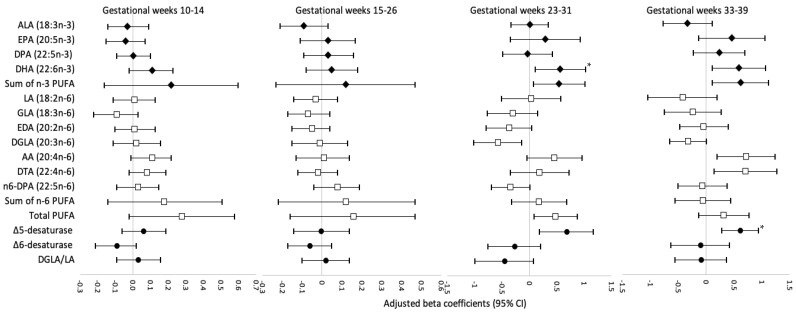
Adjusted beta coefficients for neonatal length, cm, in association with individual and subclasses of maternal plasma phospholipid PUFAs and PUFA ratios per standard deviation increase at four visits during pregnancy. AA, arachidonic acid; ALA, alpha-linolenic acid; CI, confidence interval; DHA, docosahexaenoic acid; DGLA, dihomo-gamma-linolenic acid; DPA, docosapentaenoic acid; DTA, docosatetraenoic acid; EDA, eicosadienoic acid; EPA, eicosapentaenoic acid; FDR, false discovery rate; GDM, gestational diabetes mellitus; GLA, gamma-linolenic acid; LA, linoleic acid; PUFA, polyunsaturated fatty acid; Δ5-desaturase, AA/DGLA; Δ6-desaturase, GLA/LA; Risk estimates were adjusted for maternal age (continuous), race/ethnicity (non-Hispanic White, non-Hispanic Black, Hispanic, Asian/Pacific Islander), education (high school or less, some college/associate degree, 4-year college degree or higher), nulliparity (yes/no), pre-pregnancy body mass index (<25.0, 25.0–29.9, 30.0–34.9, 35.0–44.9 kg/m^2^), gestational weight gain up to the respective visit (continuous), gestational week at blood collection (continuous), and number of days post-delivery when neonatal length was measured (continuous)). * Significant associations after FDR-adjustment (*p* < 0.05).

**Figure 3:**
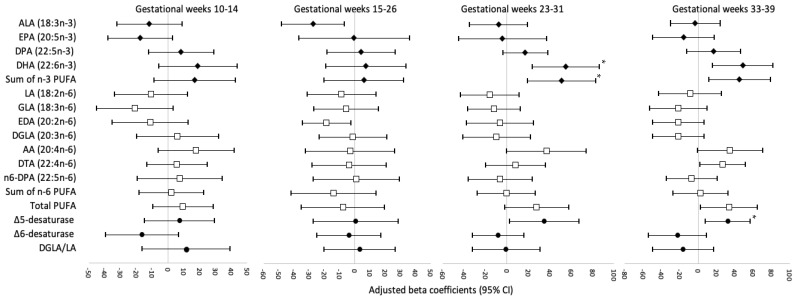
Adjusted beta coefficients for neonatal fat mass, g, in association with individual and subclasses of maternal plasma phospholipid PUFAs and PUFA ratios per standard deviation increase at four visits during pregnancy. AA, arachidonic acid; ALA, alpha-linolenic acid; CI, confidence interval; DHA, docosahexaenoic acid; DGLA, dihomo-gamma-linolenic acid; DPA, docosapentaenoic acid; DTA, docosatetraenoic acid; EDA, eicosadienoic acid; EPA, eicosapentaenoic acid; FDR, false discovery rate; GDM, gestational diabetes mellitus; GLA, gamma-linolenic acid; LA, linoleic acid; PUFA, polyunsaturated fatty acid; Δ5-desaturase, AA/DGLA; Δ6-desaturase, GLA/LA; Risk estimates were adjusted for maternal age (continuous), race/ethnicity (non-Hispanic White, non-Hispanic Black, Hispanic, Asian/Pacific Islander), education (high school or less, some college/associate degree, 4-year college degree or higher), nulliparity (yes/no), pre-pregnancy body mass index (<25.0, 25.0–29.9, 30.0–34.9, 35.0–44.9 kg/m^2^), gestational weight gain up to the respective visit (continuous), gestational week at blood collection (continuous), and number of days post-delivery when neonatal length and skinfolds were measured (continuous). * Significant associations after FDR-adjustment (*p* < 0.05).

Figure 1, Figure 2 and Figure 3 display the adjusted beta coefficients and 95% CIs for maternal plasma phospholipid PUFAs per standard deviation increase during pregnancy in association with birthweight z-score, neonatal length, and neonatal fat mass (see point estimates in Supplemental Table S2). Per SD increase in DHA (22:6*n*-3) concentrations at gestational weeks 23–31 and 33–39 was positively associated with neonatal anthropometric measures, whereas only the associations at weeks 23–31 persisted after FDR adjustment (adjusted β for birthweight z-score: 0.25, 95% CI: 0.08–0.41; neonatal length: 0.57, 0.11–1.03 cm; neonatal fat mass: 54.99, 23.57–86.42 g; all FDR < 0.05). The sum of *n*-3 PUFA levels at gestational week 23–31 was also significantly and positively associated with birthweight z-score (adjusted β = 0.23; 95% CI: 0.06, 0.40) and neonatal fat mass (adjusted β = 50.93; 95% CI: 19.07, 82.79 g), both FDR < 0.05, mostly driven by the significant associations between DHA and neonatal anthropometric measures. The level of estimated Δ5-desaturase activity per SD increase at gestational weeks 33–39 weeks was positively associated with birthweight z-score (adjusted β = 0.20; 95% CI: 0.08, 0.33), neonatal length (adjusted β = 0.61; 95% CI: 0.29, 0.94 cm), and neonatal fat mass (adjusted β = 32.59; 95% CI: 8.21, 56.96 g), all FDR < 0.05. Per SD increase in Δ5-desaturase activity at gestational weeks 23–31 was also positively associated with these three neonatal anthropometric measures; however, the associations did not persist after FDR adjustment. No other associations between individual or subclasses of PUFAs or PUFA ratios and neonatal anthropometric measures were significant after FDR adjustment. In the sensitivity analysis excluding five women who reported smoking before pregnancy, the results remained materially unchanged.

Longitudinal analysis of repeated measures of plasma phospholipid PUFAs and ratios during pregnancy in association with neonatal anthropometric measures showed overall consistent patterns with the time-specific analysis for DHA and estimated Δ5-desaturase activity. DHA levels during pregnancy were positively associated with birthweight z-score (adjusted β = 0.15; 95% CI: 0.05, 0.25) and neonatal length (adjusted β = 0.44; 95% CI: 0.12, 0.75) (Supplemental Table S3). Similarly, estimated Δ5-desaturase activity during pregnancy was positively associated with birthweight z-score (adjusted β = 0.19; 95% CI: 0.10, 0.27), neonatal length (adjusted β = 0.54; 95% CI: 0.28, 0.81), and neonatal fat mass (adjusted β = 23.96; 95% CI: 5.67, 42.24). Further, additional significant associations emerged for other individual PUFAs and ratios in relation to neonatal anthropometric measures. Specifically, per SD increase in AA (20:4*n*-6) levels during pregnancy, there was a 0.20 (95% CI: 0.10, 0.30) unit increase in birthweight z-score and 0.65 (95% CI: 0.33, 0.98) centimeter increase in neonatal length, after adjusting for covariates. Similarly, for every SD increase in DTA (22:4*n*-6) levels during pregnancy, there was a 0.18 (95% CI: 0.08, 0.28) unit increase in birthweight z-score and 0.45 (95% CI: 0.13, 0.77) centimeter increase in neonatal length, after adjusting for covariates. In contrast, per SD increase in GLA (18:3*n*-6) levels, DGLA (20:3*n*-6) levels, and estimated Δ6-desaturase activity were associated with 0.18 (95% CI: −0.28, −0.08), 0.12 (95% CI: −0.21, −0.03) and 0.17 (95% CI: −0.27, −0.07) unit decrease in birthweight z-score, respectively, after adjusting for covariates.

We also explored the potential effect modification by maternal obesity in the associations between plasma phospholipid PUFAs and neonatal anthropometric measures (Supplemental Table S4). Among women with pre-pregnancy obesity, *n*-3 PUFA DPA (22:5*n*-3) levels were positively associated with neonatal length (adjusted β = 2.99; 95% CI: 2.03, 3.94) and inversely associated with neonatal fat mass (adjusted β = −204.38; 95% CI: −311.42, −97.34) at gestational weeks 23–31 weeks. Furthermore, among *n*-6 PUFAs, LA (18:2*n*-6) levels were inversely associated with birthweight z-score (adjusted β = −0.46; 95% CI: −0.63, −0.29); EDA (20:2n6) (adjusted β = −1.6; 95% CI: −2.3, −0.87), and DGLA (20:3n6) levels (adjusted β = −1.1; 95% CI: −1.5, −0.73) were inversely associated with neonatal length (all *p* for interaction < 0.10). In contrast, AA (20:4*n*-6) levels were positively associated with birthweight z-score (adjusted β = 0.24; 95% CI: 0.08, 0.40) and neonatal length (adjusted β = 1.21; 95% CI: 0.80, 1.62) (both *p* for interaction < 0.10). Among women without pre-pregnancy obesity, DHA (22:6*n*-3) levels were positively associated with birthweight z-score (adjusted β = 0.29; 95% CI: 0.13, 0.44) (all *p* for interaction < 0.10). No other significant interaction effects between individual PUFAs or ratios with neonatal anthropometric measures were observed at gestational weeks 23–31 or other time periods.

## 4. Discussion

This longitudinal study of data from the prospective NICHD Fetal Growth Studies—Singleton Cohort provided insights into 11 individual plasma phospholipid PUFAs and 3 PUFA product-to-precursor ratios throughout gestation in relation to neonatal anthropometric measures. We found both time-specific and prospective longitudinal associations of plasma phospholipid DHA and estimated Δ5-desaturase activity with neonatal anthropometric measures. Specifically, the primarily diet-derived plasma phospholipid DHA at gestational weeks 23–31 was positively associated with birthweight z-score, neonatal length, and neonatal fat mass; however, other primarily diet-derived plasma phospholipid PUFAs, including *n*-3 EPA and *n*-6 LA, were not significantly associated with any of the neonatal anthropometric measures. Among the PUFA ratios, only the estimated Δ5-desaturase activity at gestational weeks 33–39 was positively associated with birthweight z-score, neonatal length, and neonatal fat mass.

### 4.1. Comparison with Studies on DHA and Neonatal Anthropometry

The results of previous studies on the plasma level of DHA have shown inconclusive findings regarding its associations with neonatal anthropometric measures. Rump et al. reported that lower maternal plasma phospholipid DHA levels at gestational weeks 16 weeks or earlier and umbilical cord plasma concentrations of DHA were both associated with heavier newborns in a sample of primarily Caucasian mother–infant dyads in the Netherlands [40]. In contrast, Bernard et al. found no association between maternal plasma DHA levels at gestational weeks 26–28 and birthweight among Asians [41]. We found that plasma phospholipid DHA levels at gestational weeks 23–31 were positively associated with birthweight z-score, neonatal length, and neonatal fat mass, suggesting that higher maternal plasma phospholipid DHA levels in late pregnancy may stimulate fetal growth among this multiracial/ethnic population of pregnant women with low-risk obstetrical profiles. The inconsistent findings could be attributable to differences in population characteristics, time window of exposure measurement, and varied degrees of covariate adjustment.

When evaluated longitudinally throughout pregnancy, plasma phospholipid DHA levels were no longer associated with neonatal fat mass, which may reflect an averaged-out effect due to the lack of associations between DHA at other periods of pregnancy (gestational weeks 10–14, 15–26, and 33–39) and neonatal fat mass. Given that fetal adipose depot tends to accumulate particularly in late pregnancy [42], the time-specific and longitudinal approaches suggest that 23–31 weeks of gestation could be a sensitive time window of exposure to DHA in utero in relation to fetal growth.

### 4.2. Comparison with Studies on Estimated Δ5-Desaturase Activity and Neonatal Anthropometry

We observed positive associations, from both time-specific and longitudinal analyses, between estimated Δ5-desaturase activity levels in late pregnancy (gestational weeks 33–39) and higher birthweight z-score, neonatal length, and neonatal fat mass. A recent study in Japan reported that the maternal Δ5-desaturase index at gestational weeks 24–30 did not vary among adequate-, small-, and large-for-gestational-age infants, whereas the cord blood erythrocyte Δ5-desaturase index was higher among small- versus adequate-for-gestational-age infants [43]. The different findings could be attributed to differences in study population characteristics, with a much lower median pre-pregnancy BMI in Japanese women compared to our study sample (20.5–21.9 vs. 24.6 kg/m^2^). Further, given that fetal sources of DHA are mainly through placenta-mediated transfer, these findings suggest that small-for-gestational-age infants may be aggressively synthesizing DHA based on the enhanced Δ5-desaturase activity to meet developmental needs, highlighting the important role of DHA and Δ5-desaturase in fetal growth and development.

Although some studies reported that concentrations of *n*-6 PUFA were inversely associated with birthweight [44,45], we did not find any significant associations of plasma phospholipid *n*-6 PUFA levels with neonatal growth in our time-specific analysis. Furthermore, after stratifying by obesity status, more *n*-6 PUFAs showed significant associations with neonatal anthropometric measures, highlighting that pre-pregnancy obesity status may be a potential effect modifier for the role of maternal *n*-6 PUFA levels in fetal growth. Monthé-Drèze et al. showed that women with pre-pregnancy obesity had higher concentrations of *n*-6 PUFAs and an attenuated response to *n*-3 PUFA supplementation [46]. The Dutch Generation R cohort also reported higher plasma *n*-6 PUFA levels around 20.5 weeks of gestation among women with pre-pregnancy obesity compared to women without pre-pregnancy obesity [47]. Notably, *n*-6 PUFAs are considered more proinflammatory compared to *n*-3 PUFAs [48,49]. The optimal balance of *n*-3 and *n*-6 PUFAs has been shown to reduce obesity-related inflammation [50,51]. Future investigations on the potential interplay among plasma phospholipid PUFAs, maternal obesity status, and inflammatory markers are warranted to improve our understanding of the obesity-specific associations between PUFAs and neonatal anthropometric measures.

### 4.3. Biological Plausibility and Implications

The biological mechanisms underlying the positive associations of maternal DHA and estimated Δ5-desaturase activity with neonatal anthropometric measurements among a sample of low-risk pregnant women remain to be elucidated. Plasma phospholipid DHA is primarily derived from dietary sources, such as oily fish [52]. In animal models, DHA supplementation can ameliorate the altered lung development due to intrauterine growth restriction [53]. In clinical trials, DHA supplementation has been positively linked to longer gestation duration and larger infant size [54], consistent with our observational data on newborn size. Δ5-Desaturase is one of the key enzymes that regulate the metabolism of PUFAs in humans and is necessary for DHA synthesis [52]. Our findings suggest that the enhanced activity of Δ5-desaturase (DGLA to AA) may stimulate fetal growth among our study population of low-risk pregnant women. Our results also indicate that late pregnancy may be a critical time window for the role of plasma phospholipid DHA and Δ5-desaturase in fetal growth. Indeed, fetal weight increases substantially and fetal brain growth accelerates in late pregnancy [55,56].

### 4.4. Strengths and Limitations

Our study has notable strengths. First, the prospective and longitudinal data analysis at four timepoints allowed examination of the temporal relationships between plasma phospholipid PUFA levels and enzyme activity in early to late pregnancy and neonatal anthropometric measures. Second, the objective measurement of plasma phospholipid PUFA levels allowed us to assess the associations of individual and subclasses of circulating PUFAs with measures of neonatal anthropometry. Therefore, our findings may shed light on previous inconsistent inferences concerning dietary intakes (including both foods and supplementation) of PUFAs in relation to neonatal size, which has inevitably been subject to measurement errors of dietary assessment via subjective reports. Last, our study sample was drawn from a socio-demographically diverse cohort of women across the United States, increasing the generalizability of our findings.

Several potential limitations merit discussion. Despite the significant associations observed between individual PUFAs and neonatal anthropometric measures, we cannot exclude the possibility of the relatively modest sample size causing underestimation of the significance of true associations due to statistical power. A larger sample size in future studies is needed to validate our findings. The generalization of our findings to women with higher-risk obstetrical profiles remains to be established; however, inclusion of overall healthy women may minimize reverse causality and residual confounding due to preexisting complications and unhealthy behaviors. Given that concentrations of plasma phospholipid PUFAs are a function of exogenous and endogenous sources, we cannot disentangle the specific contributions of exogenous (e.g., dietary intakes including both foods and supplementation) versus endogenous factors (e.g., genetics and biochemistry). However, the primary goal of the present study was to examine the overall circulating plasma PUFA status in association with neonatal anthropometrics. Future research examining the specific contributions of various sources to circulating levels of individual PUFAs is warranted. Finally, our findings are based on an observational study within a prospective cohort of pregnant women; further intervention studies are warranted to confirm our findings and the causal relationship.

## 5. Conclusions

Our findings suggest potentially important roles of plasma phospholipid DHA and Δ5-desaturase activity in fetal growth and development. Our findings also provide new insights into the specific time window during which PUFAs may particularly impact fetal growth, with neonatal anthropometrics as surrogate measures. More significant associations of plasma phospholipid PUFAs with neonatal anthropometric measures were particularly evident in late pregnancy (gestational weeks 23–31 and 33–39) compared to earlier gestational windows, suggesting that noncarbohydrate fuels of lipids in this sensitive time window may be particularly critical for fetal growth and development. Future research in other populations is needed to confirm our findings.

## Figures and Tables

**Table 1:** Study sample characteristics according to weighting in the NICHD Fetal Growth Studies—Singleton Cohort.

	Unweighted Overall (*n* = 2802)	Weighted Biomarker Sample (*n* = 321) ^1,2,3^
Age, years, median (interquartile range)	28.0 (24.0, 32.0)	27.6 (23.2, 31.9)
Race/ethnicity, %		
Non-Hispanic White	26.8	30.9 (23.3, 38.6)
Non-Hispanic Black	27.9	23.3 (15.6, 31.0)
Hispanic	28.7	27.2 (21.2, 33.2)
Asian/Pacific Islander	16.7	18.5 (13.4, 23.7)
Education, %		
High school or less	29.9	25.1 (17.8, 32.4)
Some college/associate degree	30.3	35.2 (27.7, 42.7)
College or higher	39.7	39.8 (32.3, 47.3)
Nulliparous	47.1	51.1 (43.3, 58.9)
Pre-pregnancy body mass index, kg/m^2^, %		
19.0–24.9	55.9	51.7 (43.9, 59.6)
25.0–29.9	27.4	33.1 (25.3, 40.8)
30.0–34.9	10.7	7.2 (3.5, 10.9)
35.0–44.9	6.0	8.0 (4.2, 11.8)
Gestational weight gain, kg, median (interquartile range)		
10–14 weeks	1.9 (0.2, 3.8)	1.9 (0.4, 3.2)
15–26 weeks	4.6 (2.3, 7.3)	4.5 (1.8, 6.8)
23–31 weeks	8.6 (5.9, 11.9)	8.7 (5.9, 11.4)
33–39 weeks	13.5 (10.0, 16.9)	13.6 (10.9, 17.2)
Total	12.2 (8.6, 16.0)	12.3 (8.5, 15.6)
Gestational diabetes, %	3.8	3.9 (2.9, 4.8)
Preeclampsia, %	3.5	2.2 (0, 4.5)
Preterm delivery, <37 gestational weeks, %	5.5	9.1 (3.6, 14.5)
Gestational age at delivery, weeks, median (interquartile range)	39.4 (38.7, 40.3)	39.3 (38.6, 40.2)
Neonatal anthropometric measurements		
Birthweight z-score, median (interquartile range)	−0.1 (−0.7, 0.5)	−0.1 (−0.7, 0.5)
Neonatal length, cm, median (interquartile range)	50.0 (48.5, 51.8)	50.5 (48.4, 51.8)
Neonatal fat mass, g, median (interquartile range)	401.6 (300.3, 515.3)	417.5 (329.0, 530.3)

^1^ Biomarker sample selected for a case–control study of gestational diabetes nested within the NICHD Fetal Growth Studies—Singleton Cohort. The sample included 107 GDM cases and 214 non-GDM controls matched 2:1 on age, race/ethnicity, and gestational week at blood collection. ^2^ Descriptive statistics for variables after applying the sampling probability weight as specified in the methods. Values are presented as weighted median (interquartile range) for continuous variables and weighted percentage and 95% confidence intervals. ^3^ Values are presented as weighted median (interquartile range) for continuous variables and weighted percentage and 95% CI for categorical variables.

**Table 2:** Mean (interquartile range) of unweighted plasma phospholipid polyunsaturated fatty acids concentrations and fatty acids ratios by gestational weeks during pregnancy (*n* = 333).

	10–14 Weeks	15–26 Weeks	23–31 Weeks	33–39 Weeks	*p*-for-Trend ^1^
***n*-3 PUFA**					
18:3*n*-3 (alpha-linolenic acid, ALA)	0.22 (0.17, 0.26)	0.25 (0.20, 0.29)	0.26 (0.20, 0.30)	0.24 (0.18, 0.29)	<0.0001
20:5*n*-3 (eicosapentaenoic acid, EPA)	0.33 (0.20, 0.41)	0.19 (0.14, 0.20)	0.31 (0.17, 0.38)	0.28 (0.16, 0.35)	<0.0001
22:5*n*-3 (docosapentaenoic acid, DPA)	0.69 (0.55, 0.81)	0.63 (0.50, 0.74)	0.60 (0.48, 0.68)	0.52 (0.41, 0.60)	<0.0001
22:6*n*-3 (docosahexaenoic acid, DHA)	4.23 (3.26, 4.94)	4.11 (3.14, 4.94)	4.06 (3.21, 4.90)	3.92 (3.04, 4.64)	0.0571
Sum of *n*-3 PUFA ^2^	5.46 (4.44, 6.30)	5.18 (4.04, 6.13)	5.22 (4.22, 6.12)	4.96 (3.96, 5.69)	0.0007
***n*-6 PUFA**					
18:2*n*-6 (linoleic acid, LA)	20.60 (18.76, 22.37)	21.65 (19.90, 23.33)	21.73 (19.66, 23.50)	22.45 (20.88, 24.08)	<0.0001
18:3*n*-6 (gamma-linoleic acid, GLA)	0.08 (0.06, 0.09)	0.08 (0.05, 0.09)	0.07 (0.05, 0.09)	0.07 (0.05, 0.09)	0.0514
20:2*n*-6 (eicosadienoic acid, EDA)	0.51 (0.44, 0.57)	0.52 (0.44, 0.58)	0.50 (0.43, 0.55)	0.44 (0.39, 0.49)	<0.0001
20:3*n*-6 (dihomo-gamma-linolenic acid, DGLA)	3.58 (2.91, 4.15)	3.56 (2.97, 4.08)	3.59 (3.02, 4.05)	3.29 (2.86, 3.76)	0.0015
20:4*n*-6 (arachidonic acid, AA)	11.22 (9.63, 12.53)	10.35 (8.78, 11.85)	9.98 (8.33, 11.26)	9.77 (8.60, 11.04)	<0.0001
22:4*n*-6 (docosatetraenoic acid, DTA)	0.49 (0.34, 0.61)	0.29 (0.21, 0.33)	0.48 (0.34, 0.59)	0.45 (0.32, 0.56)	<0.0001
22:5*n*-6 (docosapentaenoic acid, n6-DPA)	0.54 (0.38, 0.66)	0.53 (0.35, 0.64)	0.59 (0.43, 0.72)	0.59 (0.43, 0.71)	0.003
Sum of *n*-6 PUFA ^3^	37.02 (35.46, 38.70)	36.98 (35.36, 38.69)	36.96 (35.33, 38.83)	37.06 (35.73, 38.72)	0.9405
Total PUFA ^4^	42.48 (41.48, 43.86)	42.15 (40.93, 43.76)	42.16 (41.06, 43.58)	42.02 (41.03, 43.42)	0.0114
**PUFA ratios**					
Δ5-desaturase (20:4*n*-6/20:3*n*-6)	3.39 (2.47, 3.97)	3.09 (2.31, 3.67)	2.96 (2.17, 3.47)	3.14 (2.46, 3.60)	0.0001
Δ6-desaturase (18:3*n*-6/18:2*n*-6)	0.004 (0.0027, 0.005)	0.0036 (0.0024, 0.0044)	0.004 (0.0023, 0.004)	0.0034 (0.0021, 0.0041)	<0.0001
DGLA/LA (20:3*n*-6/18:2*n*-6)	0.18 (0.14, 0.21)	0.17(0.13, 0.19)	0.17 (0.14, 0.19)	0.15 (0.12, 0.17)	<0.0001

PUFA, polyunsaturated fatty acid; ^1^ obtained by Wilcoxon test; ^2^ sum of *n*-3 PUFA = ALA + EPA + DPA + DHA; ^3^ sum of *n*-6 PUFA = LA + GLA + EDA + EGLA + AA + DTA + *n*-6-DPA; ^4^ sum of *n*-3 PUFA and *n*-6 PUFA.

## Data Availability

Data described in the manuscript, code book, and analytic code will be available upon request pending application and approval of a data-sharing agreement.

## Supplementary Materials

The following are available online at: https://www.mdpi.com/article/10.3390/nu14030592/s1, Table S1: Study sample characteristics according to weighting in the NICHD Fetal Growth Studies—Singleton Cohort; Table S2: Adjusted beta coefficients for neonatal birth size and body composition in association with individual and subclasses of maternal plasma phospholipid PUFAs and PUFA ratios per standard deviation increase during pregnancy; Table S3: Adjusted beta coefficients for neonatal birth size and body composition in association with longitudinal analysis of repeated measures of individual and subclasses of maternal plasma phospholipid PUFAs and PUFA ratios per standard deviation increase across pregnancy (*n* = 333); Table S4: Adjusted beta coefficients (95% CI) for neonatal birth size and body composition in association with individual and subclasses of maternal plasma phospholipid PUFAs and PUFA ratios per standard deviation during gestational weeks of 23-31 stratified by pre-pregnancy obesity status.
