# Supplementary material for: Longitudinal Associations of Plasma Phospholipid Fatty Acids in Pregnancy with Neonatal Anthropometry: Results from the NICHD Fetal Growth Studies—Singleton Cohort
Source: Nutrients. 2022 Jan 29;14(3):592. doi: 10.3390/nu14030592 (PMC8840515; doi:10.3390/nu14030592)
Supplement: Supplementary file 1 [file nutrients-14-00592-s001.zip › nutrients-1563735-supplementary.pdf]

Supplemental Table S1. Study sample characteristics according to weighting in the NICHD Fetal Growth Studies-Singleton Cohort

	Unweighted overall (n=2,802)	Unweighted biomarker sample (n=321)[1]
Age, years, median (interquartile range)	28.0 (24.0, 32.0)	30.3 (26.2, 34.2)
Race/ethnicity, %		
Non-Hispanic White	26.8	23.4
Non-Hispanic Black	27.9	14.0
Hispanic	28.7	38.3
Asian/Pacific Islander	16.7	24.3
Education, %		
High school or less	29.9	25.2
Some college/associate degree	30.3	36.5
College or higher	39.7	38.3
Nulliparous	47.1	44.9
Pre-pregnancy body mass index, kg/m^2, %		
19.0 - 24.9	55.9	48.6
25.0 - 29.9	27.4	30.8
30.0 - 34.9	10.7	10.9
35.0 - 44.9	6.0	9.7
Gestational weight gain, kg, median (interquartile range)		
10-14 weeks	1.9 (0.2, 3.8)	2.2 (0.4, 3.7)
15-26 weeks	4.6 (2.3, 7.3)	4.5 (2.2, 6.9)
23-31 weeks	8.6 (5.9, 11.9)	8.4 (5.9, 11.5)
33-39 weeks	13.5 (10.0, 16.9)	13.0 (9.6, 16.9)
Total	12.2 (8.6, 16.0)	11.9 (8.4, 15.6)
Gestational diabetes, %	3.8	33.3
Preeclampsia, %	3.5	3.1
Preterm delivery, <37 gestational weeks, %	5.5	6.9
Gestational age at delivery, weeks, median (interquartile range)	39.4 (38.7, 40.3)	39.3 (38.7, 40.1)
Neonatal anthropometric measurements		
Birthweight z-score, median (interquartile range)	-0.1 (-0.7, 0.5)	0.0 (-0.6, 0.7)
Neonatal length, cm, median (interquartile range)	50.0 (48.5, 51.8)	50.4 (48.6, 52.0)
Neonatal fat mass, g, median (interquartile range)	401.6 (300.3, 515.3)	425.3 (332.7, 545.7)

[1]Biomarker sample selected for a case-control study of gestational diabetes nested within the NICHD Fetal Growth Studies-Singleton Cohort. The sample included 107 GDM cases and 214 non-GDM controls matched 2:1 on age, race/ethnicity, and gestational week of blood collection.

Supplemental Table S2. Adjusted beta coefficients for neonatal birth size and body composition in association with individual and subclasses of maternal plasma phospholipid PUFAs and PUFA ratios per standard deviation increase during pregnancy[1]

	Visit[2]	Birthweight z-score	Neonatal length (cm)	Neonatal fat mass (g)
**n-3 PUFA**				
ALA (18:3n-3)	0	-0.03 (-0.14, 0.09)	0.002 (-0.35, 0.36)	-11.53 (-32.16, 9.10)
	1	-0.09 (-0.21, 0.03)	0.06 (-0.27, 0.38)	-27.33 (-47.99, -6.66)
	2	-0.02 (-0.16, 0.11)	0.01 (-0.33, 0.35)	-7.73 (-35.00, 19.53)
	4	-0.07 (-0.21, 0.08)	-0.33 (-0.77, 0.11)	-2.83 (-29.80, 24.14)
EPA (20:5n-3)	0	-0.04 (-0.15, 0.07)	-0.11 (-0.43, 0.20)	-17.33 (-37.73, 3.07)
	1	0.03 (-0.11, 0.17)	0.10 (-0.21, 0.41)	-0.24 (-36.83, 36.35)
	2	0.01 (-0.15, 0.18)	0.30 (-0.34, 0.93)	-3.98 (-44.93, 36.97)
	4	-0.06 (-0.21, 0.09)	0.47 (-0.13, 1.06)	-15.66 (-49.35, 18.02)
DPA (22:5n-3)	0	0.004 (-0.09, 0.10)	-0.17 (-0.50, 0.16)	8.44 (-12.06, 28.95)
	1	0.03 (-0.09, 0.16)	0.05 (-0.35, 0.45)	4.35 (-18.01, 26.70)
	2	0.06 (-0.07, 0.19)	-0.03 (-0.48, 0.43)	17.34 (-3.86, 38.53)
	4	0.10 (-0.07, 0.26)	0.24 (-0.22, 0.70)	17.00 (-12.52, 46.52)
DHA (22:6n-3)	0	0.11 (-0.02, 0.23)	0.27 (-0.11, 0.65)	19.03 (-5.64, 43.71)
	1	0.05 (-0.08, 0.18)	0.11 (-0.23, 0.45)	7.53 (-18.91, 33.97)
	2	0.25 (0.08, 0.41)[3]	0.57 (0.11, 1.03)[3]	54.99 (23.57, 86.42)[3]
	4	0.21 (0.05, 0.37)	0.59 (0.11, 1.07)	49.16 (16.19, 82.13)
Sum of n-3 PUFA	0	0.10 (-0.03, 0.22)	0.22 (-0.16, 0.60)	17.01 (-8.60, 42.62)
	1	0.05 (-0.09, 0.18)	0.12 (-0.23, 0.47)	6.22 (-20.06, 32.51)
	2	0.23 (0.06, 0.40)[3]	0.55 (0.08, 1.02)	50.93 (19.07, 82.79)[3]
	4	0.19 (0.02, 0.36)	0.62 (0.11, 1.12)	45.56 (11.63, 79.48)
**n-6 PUFA**				
LA (18:2n-6)	0	0.01 (-0.11, 0.13)	0.17 (-0.22, 0.55)	-10.58 (-33.71, 12.55)
	1	-0.03 (-0.14, 0.08)	-0.004 (-0.37, 0.36)	-8.51 (-31.32, 14.30)
	2	-0.01 (-0.16, 0.14)	0.04 (-0.50, 0.58)	-15.74 (-43.16, 11.68)
	4	-0.02 (-0.20, 0.16)	-0.42 (-1.04, 0.20)	-8.58 (-43.13, 25.97)
GLA (18:3n-6)	0	-0.09 (-0.22, 0.03)	-0.22 (-0.55, 0.11)	-20.71 (-44.78, 3.36)
	1	-0.07 (-0.17, 0.04)	-0.30 (-0.67, 0.08)	-5.66 (-26.99, 15.68)
	2	-0.16 (-0.30, -0.02)	-0.30 (-0.76, 0.15)	-11.77 (-36.40, 12.86)
	4	-0.2 (-0.40, -0.01)	-0.23 (-0.74, 0.27)	-21.41 (-53.07, 10.26)
EDA (20:2n-6)	0	0.01 (-0.10, 0.13)	0.12 (-0.27, 0.50)	-10.97 (-34.94, 12.99)
	1	-0.05 (-0.15, 0.04)	-0.09 (-0.40, 0.22)	-18.47 (-34.47, -2.48)
	2	-0.06 (-0.23, 0.11)	-0.37 (-0.79, 0.05)	-6.37 (-37.63, 24.88)
	4	-0.10 (-0.25, 0.05)	-0.04 (-0.47, 0.40)	-21.38 (-49.48, 6.82)
DGLA (20:3n-6)	0	0.02 (-0.11, 0.16)	-0.19 (-0.61, 0.24)	6.22 (-19.79, 32.24)
	1	-0.01 (-0.15, 0.13)	-0.16 (-0.59, 0.27)	-1.09 (-23.46, 21.28)
	2	-0.13 (-0.30, 0.03)	-0.57 (-1.01, -0.14)	-9.68 (-41.27, 21.90)
	4	-0.14 (-0.28, 0.01)	-0.32 (-0.65, 0.004)	-21.14 (-49.18, 6.91)
AA (20:4n-6)	0	0.11 (-0.01, 0.22)	0.12 (-0.26, 0.50)	17.92 (-5.97, 41.81)
	1	0.01 (-0.13, 0.14)	0.20 (-0.14, 0.55)	-2.84 (-32.27, 26.58)
	2	0.12 (-0.02, 0.27)	0.46 (-0.04, 0.97)	36.98 (-0.28, 74.25)
	4	0.18 (0.03, 0.32)	0.72 (0.20, 1.24)	35.07 (-0.36, 70.51)

DTA (22:4n-6)	0	0.08 (-0.02, 0.19)	0.04 (-0.27, 0.35)	5.80 (-13.38, 24.98)
	1	-0.02 (-0.12, 0.08)	0.06 (-0.26, 0.38)	-3.44 (-27.81, 20.94)
	2	0.07 (-0.07, 0.22)	0.19 (-0.34, 0.73)	8.11 (-19.95, 36.17)
	4	0.20 (0.07, 0.33)	0.71 (0.15, 1.27)	26.87 (2.24, 51.50)
n6-DPA (22:5n-6)	0	0.03 (-0.09, 0.15)	-0.05 (-0.42, 0.31)	7.66 (-19.23, 34.56)
	1	0.08 (-0.04, 0.19)	0.01 (-0.38, 0.40)	1.10 (-27.34, 29.54)
	2	-0.07 (-0.19, 0.05)	-0.34 (-0.69, 0.01)	-6.15 (-36.16, 23.87)
	4	-0.05 (-0.18, 0.09)	-0.06 (-0.50, 0.38)	-6.72 (-34.47, 21.02)
Sum of n-6 PUFA	0	0.09 (-0.02, 0.19)	0.18 (-0.14, 0.51)	2.30 (-17.98, 22.57)
	1	-0.03 (-0.16, 0.11)	0.12 (-0.22, 0.47)	-13.75 (-41.68, 14.19)
	2	0.04 (-0.11, 0.20)	0.18 (-0.32, 0.69)	-0.51 (-27.68, 26.65)
	4	0.06 (-0.11, 0.23)	-0.05 (-0.55, 0.44)	2.81 (-26.97, 32.59)
**Total PUFA**	0	0.12 (0.03, 0.22)	0.28 (-0.02, 0.58)	9.62 (-9.40, 28.64)
	1	0.002 (-0.13, 0.13)	0.16 (-0.16, 0.47)	-7.64 (-35.17, 19.89)
	2	0.15 (0.02, 0.29)	0.48 (0.09, 0.88)	27.89 (-1.86, 57.63)
	4	0.17 (0.03, 0.31)	0.32 (-0.13, 0.77)	33.85 (2.91, 64.80)
**PUFA ratios**				
Δ5-desaturase	0	0.06 (-0.06, 0.19)	0.22 (-0.12, 0.57)	7.44 (-14.57, 29.46)
	1	-0.004 (-0.14, 0.14)	0.24 (-0.13, 0.61)	0.91 (-27.13, 28.94)
	2	0.18 (0.02, 0.34)	0.68 (0.19, 1.17)	34.83 (2.52, 67.14)
	4	0.20 (0.08, 0.33)[3]	0.61 (0.29, 0.94)[4]	32.59 (8.21, 56.96)[3]
Δ6-desaturase	0	-0.09 (-0.21, 0.02)	-0.26 (-0.61, 0.09)	-16.32 (-39.44, 6.81)
	1	-0.06 (-0.17, 0.05)	-0.31 (-0.70, 0.08)	-3.74 (-24.84, 17.35)
	2	-0.14 (-0.28, -0.01)	-0.27 (-0.75, 0.21)	-8.25 (-32.26, 15.76)
	4	-0.20 (-0.37, -0.02)	-0.10 (-0.63, 0.42)	-22.33 (-54.08, 9.42)
DGLA/LA	0	0.03 (-0.09, 0.16)	-0.20 (-0.66, 0.26)	11.65 (-16.05, 39.35)
	1	0.02 (-0.10, 0.14)	-0.11 (-0.51, 0.30)	3.40 (-20.03, 26.84)
	2	-0.09 (-0.25, 0.06)	-0.45 (-0.99, 0.08)	-0.58 (-32.08, 30.92)
	4	-0.11 (-0.26, 0.04)	-0.09 (-0.55, 0.37)	-16.35 (-49.59, 16.89)

[1]Adjusted for maternal age (continuous), race/ethnicity (non-Hispanic white, non-Hispanic black, Hispanic, Asian), education (high school or less, some college/associate degree, 4-year college degree or higher), nulliparity (yes/no), pre-pregnancy body mass index (<25.0, 25.0-29.9, 30.0-34.9, 35.0-44.9 kg/m^2), gestational weight gain up to the respective visit (continuous), gestational week at blood collection (continuous), gestational age at delivery (continuous), and postnatal days at neonatal assessment (continuous; all models except for the one on birthweight).
[2]Visits 0, 1, 2, 4: Gestational weeks 10-14, 15-26, 23-31, 33-39, respectively.
[3]*P*< 0.05 after false-discovery rate correction.
[4]*P*< 0.01 after false-discovery rate correction.

Supplemental Table S3. Adjusted beta coefficients for neonatal birth size and body composition in association with longitudinal analysis of repeated measures of individual and subclasses of maternal plasma phospholipid PUFAs and PUFA ratios per standard deviation increase across pregnancy (n = 333).[1,2]

	Birthweight z-score	Neonatal length (cm)	Neonatal fat mass (g)
**n-3 PUFA**			
ALA (18:3n-3)	-0.10 (-0.20, -0.00)	-0.33 (-0.63, -0.03)	-8.12 (-27.85, 11.61)
EPA (20:5n-3)	-0.04 (-0.15, 0.07)	0.30 (-0.04, 0.63)	-18.20 (-40.22, 3.81)
DPA (22:5n-3)	0.04 (-0.06, 0.14)	0.12 (-0.18, 0.42)	6.20 (-13.82, 26.21)
DHA (22:6n-3)	0.15 (0.05, 0.25)[3]	0.44 (0.12, 0.75)[3]	24.74 (4.46, 45.03)

Sum of n-3 PUFA	0.13 (0.03, 0.23)	0.43 (0.11, 0.74)[3]	21.07 (0.77, 41.36)
**n-6 PUFA**			
LA (18:2n-6)	-0.01 (-0.12, 0.10)	-0.22 (-0.56, 0.11)	-7.56 (-29.30, 14.19)
GLA (18:3n-6)	-0.18 (-0.28, -0.08)[4]	-0.34 (-0.66, -0.02)	-23.39 (-43.78, -2.99)
EDA (20:2n-6)	-0.07 (-0.18, 0.03)	-0.00 (-0.33, 0.33)	-17.48 (-38.82, 3.87)
DGLA (20:3n-6)	-0.12 (-0.21, -0.03)[3]	-0.30 (-0.57, -0.03)	-13.48 (-31.55, 4.59)
AA (20:4n-6)	0.20 (0.10, 0.30)[4]	0.65 (0.33, 0.98)[4]	30.78 (6.72, 54.85)
DTA (22:4n-6)	0.18 (0.08, 0.28)[3]	0.45 (0.13, 0.77)[3]	23.13 (1.18, 45.07)
n6-DPA (22:5n-6)	-0.03 (-0.13, 0.06)	-0.11 (-0.40, 0.17)	-2.57 (-22.59, 17.45)
Sum of n-6 PUFA	0.09 (-0.01, 0.19)	0.13 (-0.18, 0.43)	3.82 (-16.07, 23.72)
**Total PUFA**	0.17 (0.07, 0.26)[4]	0.40 (0.08, 0.71)	17.27 (-3.61, 38.15)
**PUFA ratios**			
Δ5-desaturase (20:4n-6/20:3n-6)	0.19 (0.10, 0.27)[5]	0.54 (0.28, 0.81)[5]	23.96 (5.67, 42.24)[3]
Δ6-desaturase (18:3n-6/18:2n-6)	-0.17 (-0.27, -0.07)[4]	-0.26 (-0.56, 0.05)	-22.92 (-42.77, -3.07)
DGLA/LA (20:3n-6/18:2n-6)	-0.10 (-0.19, -0.00)	-0.14 (-0.43, 0.15)	-9.47 (-28.93, 9.99)

[1]Adjusted for maternal age (continuous), race/ethnicity (non-Hispanic white, non-Hispanic black, Hispanic, Asian), education (high school or less, some college/associate degree, 4-year college degree or higher), nulliparity (yes/no), pre-pregnancy body mass index (<25.0, 25.0-29.9, 30.0-34.9, 35.0-44.9 kg/m^2), gestational weight gain up to the respective visit (continuous), gestational week at blood collection (continuous), gestational age at delivery (continuous), and postnatal days at neonatal assessment (continuous; all models except for the one on birthweight); [2]Visits 0, 1, 2, 4: Gestational weeks 10-14, 15-26, 23-31, 33-39, respectively; [3]$P < 0.05$ after false-discovery rate correction; [4]$P < 0.01$ after false-discovery rate correction; [5]$P < 0.001$ after false-discovery rate correction.

Supplemental Table S4. Adjusted beta coefficients (95% CI) for neonatal birth size and body composition in association with individual and subclasses of maternal plasma phospholipid PUFAs and PUFA ratios per standard deviation during gestational weeks of 23-31 stratified by pre-pregnancy obesity status[1]

	Birthweight z-score		Neonatal length (cm)		Neonatal fat mass (g)	
	Non-obese	Obese	Non-obese	Obese	Non-obese	Obese
**n-3 PUFA**						
18:3n-3 (alpha-linolenic acid, ALA)	-0.02 (-0.16, 0.13)	-0.40 (-0.86, 0.05)	-0.07 (-0.44, 0.30)	-0.03 (-1.5, 1.39)	-4.80 (-34.87, 25.28)	36.79 (-58.42, 132.01)
20:5n-3 (eicosapentaenoic acid, EPA)	0.09 (-0.10, 0.29)	-0.27 (-0.51, -0.04)	0.47 (-0.18, 1.12)	-0.42 (-1.1, 0.25)	16.47 (-35.01, 67.96)	-44.73 (-121.72, 32.26)
22:5n-3 (docosapentaenoic acid, DPA)	0.09 (-0.03, 0.21)	0.26 (-0.12, 0.63)	-0.02 (-0.42, 0.39)[*]	2.99 (2.03, 3.94)[2*]	23.49 (3.69, 43.28)[*]	-204.38 (-311.42, -97.34)[3*]
22:6n-3 (docosahexaenoic acid, DHA)	0.29 (0.13, 0.44)[3*]	0.01 (-0.54, 0.57)[*]	0.56 (0.11, 1.01)[4]	2.66 (1.49, 3.84)[2]	56.53 (26.96, 86.09)[3]	67.23 (-38.53, 172.99)
Sum of n-3 PUFA	0.28 (0.12, 0.44)	-0.09 (-0.68, 0.50)	0.55 (0.08, 1.01)	3.45 (2.09, 4.80)[2]	55.77 (25.83, 85.71)[3]	30.39 (-101.71, 162.49)
**n-6 PUFA**						
18:2n-6 (linoleic acid, LA)	-0.01 (-0.17, 0.15)[*]	-0.46 (-0.63, -0.29)[2*]	-0.02 (-0.55, 0.50)	-0.80 (-1.6, 0.05)	-15.74 (-42.14, 10.65)	-86.20 (-160.13, -12.27)
18:3n-6 (gamma-linoleic acid, GLA)	-0.16 (-0.31, -0.01)	0.17 (-0.004, 0.35)	-0.23 (-0.70, 0.24)	0.17 (-0.26, 0.61)	-14.75 (-41.14, 11.64)	46.00 (22.89, 69.10)[2]
20:2n-6 (eicosadienoic acid, EDA)	-0.04 (-0.23, 0.14)	-0.16 (-0.45, 0.13)	-0.26 (-0.69, 0.18)[*]	-1.6 (-2.3, -0.87)[2*]	-2.58 (-34.46, 29.29)	-42.95 (-101.94, 16.05)
20:3n-6 (dihomo-gamma-linolenic acid, DGLA)	-0.12 (-0.31, 0.07)	-0.02 (-0.25, 0.21)	-0.43 (-0.91, 0.05)[*]	-1.1 (-1.5, -0.73)[2*]	1.56 (-33.52, 36.63)	46.99 (-21.16, 115.13)
20:4n-6 (arachidonic acid, AA)	0.18 (0.01, 0.35)[*]	0.24 (0.08, 0.40)[4*]	0.51 (-0.02, 1.05)[*]	1.21 (0.80, 1.62)[2*]	36.26 (-2.05, 74.56)	29.48 (-21.67, 80.64)
22:4n-6 (docosatetraenoic acid, DTA)	0.13 (-0.04, 0.31)	-0.10 (-0.35, 0.14)	0.35 (-0.23, 0.92)	-0.63 (-1.3, 0.08)	23.38 (-13.06, 59.83)	-62.92 (-111.48, -14.37)
22:5n-6 (docosapentaenoic acid, n6-DPA)	-0.10 (-0.23, 0.03)	0.28 (0.01, 0.54)	-0.41 (-0.76, -0.06)	-0.91 (-2.6, 0.78)	-5.03 (-33.96, 23.89)	-40.26 (-146.10, 65.58)
Sum of n-6 PUFA	0.07 (-0.09, 0.22)	-0.55 (-0.76, -0.33)[2]	0.16 (-0.31, 0.63)	-0.10 (-1.3, 1.10)	2.15 (-24.64, 28.94)	-85.28 (-179.31, 8.76)
**Total of PUFA**	0.22 (0.09, 0.35)[3]	-0.39 (-0.56, -0.23)[2]	0.48 (0.09, 0.88)	0.58 (-0.41, 1.56)	36.65 (8.11, 65.20)	-53.77 (-132.54, 24.99)

**PUFA ratios**						
Δ5-desaturase	0.26 (0.09, 0.43)[4]	0.14 (0.04, 0.25)	0.80 (0.39, 1.21)[2]	0.82 (0.54, 1.10)[2]	27.94 (-10.35, 66.23)	-4.04 (-53.67, 45.60)
Δ6-desaturase	-0.15 (-0.29, -0.003)	0.27 (0.06, 0.48)[4]	-0.20 (-0.69, 0.29)	0.40 (-0.10, 0.90)	-9.41 (-33.90, 15.07)	60.16 (30.42, 89.90)[2]
DGLA_LA	-0.09 (-0.26, 0.09)	0.21 (-0.05, 0.47)	-0.29 (-0.86, 0.27)[*]	-1.70 (-2.50, -0.95)[2*]	9.71 (-21.58, 41.01)[*]	123.24 (46.38, 200.10)[3*]

[1]Adjusted for maternal age (continuous), race/ethnicity (non-Hispanic white, non-Hispanic black, Hispanic, Asian), education (high school or less, some college/associate degree, 4-year college degree or higher), nulliparity (yes/no), pre-pregnancy body mass index (<25.0, 25.0-29.9, 30.0-34.9, 35.0-44.9 kg/m^2), gestational weight gain up to the respective visit (continuous), gestational week at blood collection (continuous), gestational age at delivery (continuous), and postnatal days at neonatal assessment (continuous; all models except for the one on birthweight).

[2]$P < 0.001$ after false-discovery rate correction.

[3]$P < 0.01$ after false-discovery rate correction.

[4]$P < 0.05$ after false-discovery rate correction.

[*]P for interaction <0.10.
